# Human polyomavirus type six in respiratory samples from hospitalized children with respiratory tract infections in Beijing, China

**DOI:** 10.1186/s12985-015-0390-5

**Published:** 2015-10-13

**Authors:** Wen-zhi Zheng, Tian-li Wei, Fen-lian Ma, Wu-mei Yuan, Qian Zhang, Ya-xin Zhang, Hong Cui, Li-shu Zheng

**Affiliations:** Key Laboratory for Medical Virology, Ministry of Health, National Institute for Viral Disease Control and Prevention, China CDC, Beijing, 100052 China; Department of Pediatrics, Beijing Friendship Hospital, Capital Medical University, 95 Yong An St., Xi-Cheng District, Beijing, 100050 China

**Keywords:** Human polyomavirus type six, TaqMan real-time PCR, Nasopharyngeal aspirate specimen, Respiratory virus

## Abstract

**Background:**

HPyV6 is a novel human polyomavirus (HPyV), and neither its natural history nor its prevalence in human disease is well known. Therefore, the epidemiology and phylogenetic status of HPyV6 must be systematically characterized.

**Methods:**

The VP1 gene of HPyV6 was detected with an established TaqMan real-time PCR from nasopharyngeal aspirate specimens collected from hospitalized children with respiratory tract infections. The HPyV6-positive specimens were screened for other common respiratory viruses with real-time PCR assays.

**Results:**

The prevalence of HPyV6 was 1.7 % (15/887), and children ≤ 5 years of age accounted for 80 % (12/15) of cases. All 15 HPyV6-positive patients were coinfected with other respiratory viruses, of which influenza virus A (IFVA) (8/15, 53.3 %) and respiratory syncytial virus (7/15, 46.7 %) were most common. All 15 HPyV6-positive patients were diagnosed with lower respiratory tract infections, and their viral loads ranged from 1.38 to 182.42 copies/μl nasopharyngeal aspirate specimen. The most common symptoms were cough (100 %) and fever (86.7 %). The complete 4926-bp genome (BJ376 strain, GenBank accession number KM387421) was amplified and showed 100 % identity to HPyV6 strain 607a.

**Conclusions:**

The prevalence of HPyV6 was 1.7 % in nasopharyngeal aspirate specimens from hospitalized children with respiratory tract infections, as analyzed by real-time PCR. Because the coinfection rate was high and the viral load low, it was not possible to establish a correlation between HPyV6 and respiratory diseases.

**Electronic supplementary material:**

The online version of this article (doi:10.1186/s12985-015-0390-5) contains supplementary material, which is available to authorized users.

## Background

The family *Polyomaviridae* contains viruses that are among the smallest known to infect humans, in terms of both their particle sizes and genome lengths. The 40–45 nm nonenveloped icosahedral particles carry a circular double-stranded DNA genome of approximately 5000 bp, which is divided into three functional regions: the noncoding control region, the early gene region encoding the large T antigen and the small T antigen, and the late coding region, encoding capsid proteins VP1, VP2, and VP3 [1].

Human polyomaviruses (HPyVs) have not been associated with any severe acute disease in healthy humans. However, the viral proteins expressed by polyomaviruses (PyVs) can initiate the transformation and immortalization of cultured cells and cause cancer in experimental animals [2]. The HPyV family currently consists of 13 members, including JCPyV [3], BKPyV [4], WUPyV [5], KIPyV [6], MCPyV [7], HPyV6 [8], HPyV7 [8], TSPyV [9], HPyV9 [10], HPyV10 [11–13], STLPyV [14], HPyV12 [15] and NJPyV-2013 [16].

Previous studies have indicated that a number of HPyVs are associated with human diseases, such as progressive multifocal leukoencephalopathy (JCPyV), hemorrhagic cystitis (BKPyV), Merkel cell carcinoma (MCPyV), and trichodysplasia spinulosa (TSPyV) [3, 7, 9, 17–19]. A serological study demonstrated that the seroprevalence of these viruses in 1501 plasma samples from healthy adult blood donors was BKPyV (82 %), JCPyV (39 %), KIPyV (55 %), WUPyV (69 %), MCPyV isolate 350 (25 %); and MCPyV isolate 339 (42 %). The seroprevalence of all polyomaviruses in children under 21 years of age (*n* = 721) was similar to that in the adult population, suggesting that primary exposure to these viruses occurs in early childhood and seems to result in lifelong persistence [20]. Nevertheless, the natural histories of most HPyVs and their prevalence in human diseases are not yet well known.

In 2008, MCPyV was discovered by Feng et al. [7]. HPyV6 and HPyV7 were discovered with MCPyV in skin swabs from the foreheads of healthy volunteers [8]. However, later research could not demonstrate a relationship between HPyV6 and Merkel cell carcinoma or other skin diseases. A phylogenetic analysis of the complete HPyV6 genome indicated that HPyV6 shared a branch with KIPyV and WUPyV. Previous reports have shown that genomic fragments of KIPyV and WUPyV have been regularly detected in nasopharyngeal aspirates of children with respiratory tract infections (RTIs) and are suspected of a causal relationship with respiratory disease. However, the link between these PyVs and respiratory diseases remains speculative [5, 6, 21, 22].

A real-time PCR assay was established here to determine the prevalence of HPyV6 throughout a time period of 12 months (from October 2011 to September 2012). The prevalence of HPyV6 was 1.7 % (15/887). A complete HPyV6 genome was amplified, sequenced and found to be identical with a HPyV6 isolate from USA.

## Results

### Establishment and evaluation of real-time PCR assay

With the standard curve derived from serial DNA dilutions, the dynamic range of the real-time PCR assay was 10^0^–10^10^ copies/μl and the limit of detection was one copy. The coefficient of determination (*R*^*2*^ = 0.99667) showed a good linear correlation. The TaqMan-based real-time PCR assay to detect HPyV6 did not amplify any other viral pathogen, showing the excellent specificity of this assay. Four different DNA concentrations (10^5^–10^8^ copies per reaction) were repeated five times in each run. The maximum coefficient of variation was 0.66 %, which indicates good precision (data not shown).

### Epidemiology of HPyV6

**Fig. 1:**
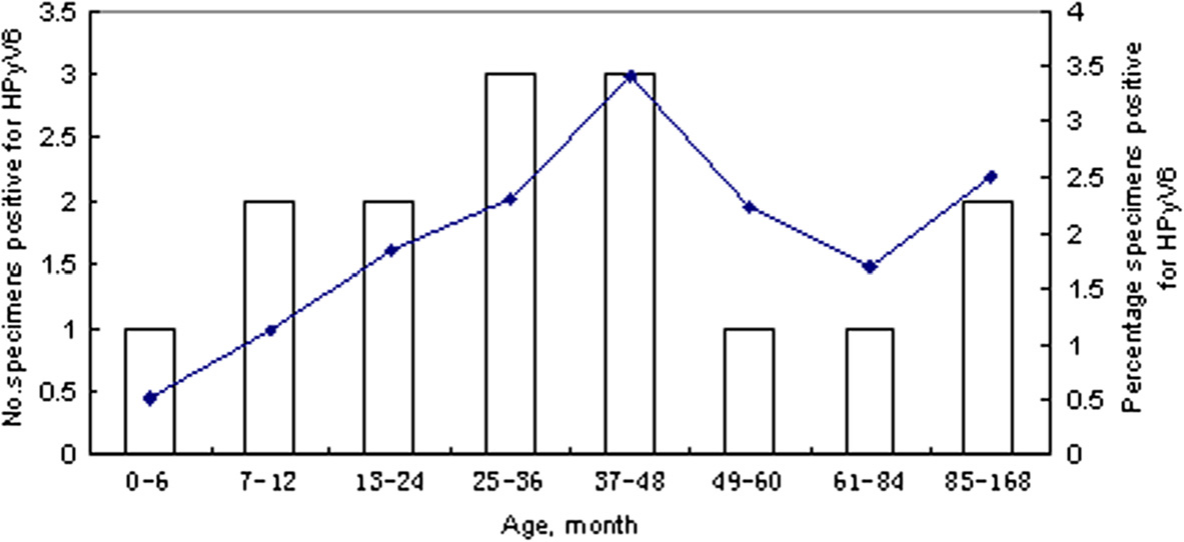
Age distribution of children infected with HPyV6

**Fig. 2:**
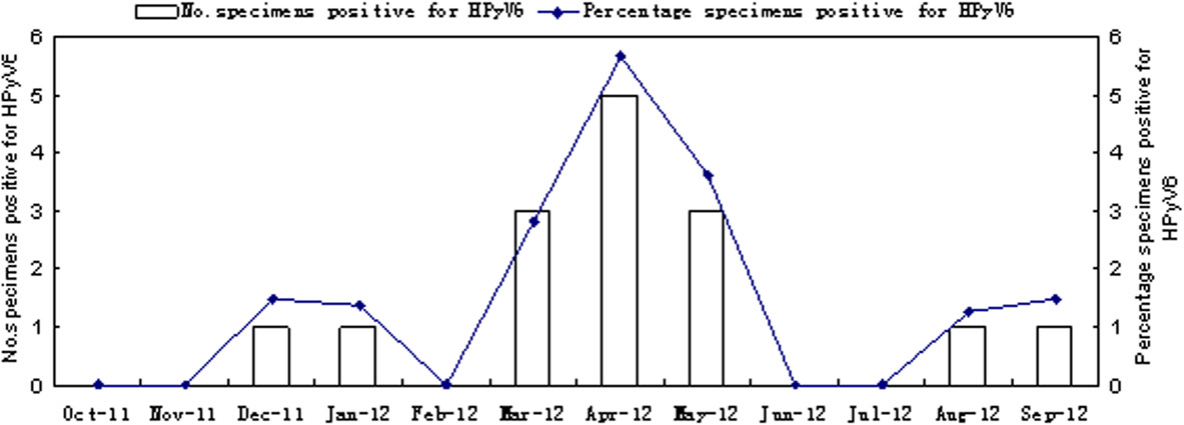
Seasonal distribution of children infected with HPyV6

A total of 887 NPA samples were obtained from 887 children with RTI. The sex ratio (male:female) was 524:363 (1.44:1) and the median age was 24 months (ranging from 3 days to 14 years). The prevalence of HPyV6 was 1.7 % (15/887) in the 887 NPA specimens tested. Of the 15 HPyV6-positive patients, eight were male (8/524, 1.53 %) and seven were female (7/363, 1.93 %), so the prevalence was similar in both sexes (*p* > 0.05). The ages of the infected patients ranged from 4 days to 13 years, and children ≤ 5 years of age accounted for 80 % (12/15) of the total HPyV6-positive children. The age distribution of the HPyV6-infected children indicated that those aged 37–48 months had the highest infection rate (3.41 %) (Fig. 1). HPyV6 was detected in every month of the study year, except February, June, July, October, and November. The majority of HPyV6 cases were detected in spring (from March 2012 to May 2012), accounting for 73.3 % (11/15) (Fig. 2), and the peak incidence (5/88, 5.68 %) occurred in April 2012.

Nucleic acids extracted from all 887 NPA samples were tested for the VP1 gene of HPyV6 with the previously established TaqMan real-time PCR. The HPyV6-positive specimens were then screened for human metapneumovirus (hMPV), respiratory syncytial virus (RSV), human bocavirus (HBoV), influenza viruses A (IFVA) and B (IFVB), parainfluenza virus types 1–4 (PIV1-4), human rhinoviruses (HRVs), adenovirus (ADV), human coronaviruses (229E, OC43, NL63, and HKU1), WUPyV, and KIPyV with real-time PCR assays. The primers and probes used in this study are listed in Additional file 1: Table S1 [23–31]. All 15 HPyV6-positive patients were coinfected with1-4 respiratory viruses. Most HPyV6-associated coinfections involved IFVA (8/15, 53.3 %) or RSV (7/15, 46.7 %) (Table 1).

**Table 1:** Codetection of HPyV6 and other common respiratory viruses

	KIPyV	WUPyV	HBoV	HRV	ADV	HMPV	RSV	IFVA	IFVB	PIV-1	PIV-2	PIV-3	PIV-4	HCoV-229E	HCoV-OC43	HCoV-HKU1	HCoV-NL63
BJ148	+					+	+					+					
BJ211	+						+										
BJ361									+						+		
BJ375		+			+		+	+									
BJ376							+		+								
BJ454								+									
BJ460				+				+								+	
BJ488					+			+							+		
BJ508		+			+		+										
BJ511						+		+									+
BJ520							+	+									
BJ537														+			
BJ556																+	
BJ767	+							+									
BJ840					+		+	+						+			

+ represents positive; *HBoV* human bocavirus; *HRV* human rhinoviruse; *ADV* adenovirus; *hMPV* human metapneumovirus; *RSV* respiratory syncytial virus; *IFVA* influenza viruses A; *IFVB* influenza viruses B; *PIV1-4*, parainfluenza virus types 1–4; HCoV 229E, OC43, NL63, *HKU1*, human coronaviruses 229E, OC43, NL63, HKU1

### Viral load and clinical characteristics of HPyV6 in children

All 15 HPyV6-positive patients were diagnosed with lower RTI, including bronchopneumonia in nine (60 %), acute bronchitis in three (20 %), bronchitis in two (13.3 %), and pneumonia in one (6.7 %). The most common symptom was cough, which occurred in all 15 patients (100 %). Other clinical presentations included fever (*n* = 13, 86.7 %), gasping (*n* = 3, 20 %), vomiting (*n* = 3, 20 %), and diarrhea (*n* = 1, 6.7 %). Among the HPyV6-positive specimens, the HPyV6 genome copies ranged from 1.38 to 182.42 copies/μl NPA on a real-time PCR assay (Table 2). The HPyV6 genome copy number was 52.07 copies/μl NPA in children suffering lower RTIs only, and was slightly higher than 29.75 copies/μl NPA in those infected with lower RTIs and other diseases, but these did not differ significantly (*p* > 0.05, Mann–Whitney *U* test).

**Table 2:** Viral loads and clinical characteristics of HPyV6-positive children with respiratory tract infections

Specimen	Sex	Age (month)	Date	Signs/symptoms	Diagnosis	Viral load in NPA (copies/μL)
BJ148	F	156	Dec-11	Fever, cough	Bronchopneumonia, congenital hypoplasia of left kidney	1.38
BJ211	M	3	Jan-12	cough, gasping, diarrhea	Bronchopneumonia, malnutrition	8.84
BJ361	F	30	Mar-12	Fever, cough, vomiting	Acute bronchitis, congenital hydronephrosis, hypokalemia, dyspepsia, oral exanthema	48.53
BJ375	F	108	Mar-12	Fever, cough	Acute bronchitis	54.95
BJ376	M	9	Mar-12	cough, gasping	Bronchitis	182.42
BJ454	M	34	Apr-12	Fever, cough	Bronchopneumonia	47.21
BJ460	M	48	Apr-12	Fever, cough, vomiting	Bronchopneumonia, ventricular premature beat	68.99
BJ488	F	60	Apr-12	Fever, cough	Acute bronchitis, acute suppurative tonsillitis	68.05
BJ508	M	36	Apr-12	Fever, cough	Bronchopneumonia, infectious mononucleosis syndrome, acute suppurative tonsillitis	29.14
BJ511	F	44	Apr-12	Fever, cough	Bronchopneumonia	21.96
BJ520	M	41	May-12	Fever, cough, vomiting	Bronchopneumonia, rhinitis	2.93
BJ537	F	22	May-12	Fever, cough	Bronchopneumonia, hepatomegaly	10.14
BJ556	F	24	May-12	Fever, cough	Mycoplasmal pneumonia	36.08
BJ767	M	84	Aug-12	Fever, cough, gasping	Bronchitis	16.55
BJ840	M	11	Sep-12	Fever, cough	Bronchopneumonia	3.34

To determine the complete HPyV6 genomic sequence, overlapping genomic fragments were amplified with nested PCR using 15 pairs of virus-specific primers (listed in Additional file 1: Table S2), and the complete 4926-bp genome was compiled with BioEdit 9.0 software. The genome sequence of HPyV6 was deposited in GenBank under accession number KM387421. BLAST analysis with the complete HPyV6 genome showed a high level of nucleic acid identity to the six full HPyV6 genomes available in GenBank. Relationsship to the HPyV6 strain 607a was 100 %.

## Discussion

HPyV6, thought to be a skin-tropic polyomavirus, was initially described in 2010. Since then, subgenomic fragments of HPyV6 DNA have been detected in a variety of specimen types, including skin, respiratory secretion samples, and various tumor samples (for instance, squamous cell carcinoma, basal cell carcinoma, and multiple myeloma) [8, 32–34]. Although HPyVs had been identified several decades ago, there are still many basic questions to be clarified, for examples, how HPyVs interact with their hosts and how to spread throughout a population. For instance, BKPyV and JCPyV are shed in the urine and transmitted via the respiratory route. No data on the immune responses of healthy individuals to the HPyVs are available. It is unknown whether the HPyVs exist in human cells with low viral replication to establish a latent state, or how the cells restrict viral replication. The answers to these questions will require more information on the biology and epidemiology of the HPyVs.

The genomes and proteins of HPyV6, one of the novel HPyVs, show little sequence homology with previously reported HPyVs (BKPyV and JCPyV). Although HPyV6 encodes a conserved, potentially carcinogenic LTAg, previous studies have shown no association between HPyV6 and tumors [1]. Furthermore, a phylogenetic analysis indicated that LTAg of HPyV6 (KM387421) is only distantly related to its homologues in other cancer-associated HPyVs. The HPyV6, WUPyV, and KIPyV strains formed a clade in the complete genome and VP1 amino acid phylogenies, whether HPyV6 also associate with respiratory infection which need more clinical and experimental evidences to support. HPyV6 has been detected in specimens from the human respiratory tract, but there are as yet insufficient epidemiological data to demonstrate a correlation between HPyV6 and respiratory disease. Because initial infections with most HPyVs occur in infancy, the prevalence of HPyV6 in NPAs from children was detected with real-time PCR.

HPyV6 displayed an overall prevalence of 1.7 % in NPA samples collected from children in a hospital in China, which is similar to its prevalence reported previously (0.5–2 %) [34, 35]. It has not been confirmed that HPyV6 infects humans via the respiratory tract, but the respiratory tract may be a possible route of transmission. In this study, HPyV6 was mainly detected in children less than 5 years of age, and the peak incidence occurred in spring. All 15 HPyV6-positive patients were coinfected with other respiratory viruses, of which IFVA and RSV were the most common. The HPyV6-positive patients were diagnosed with lower RTIs, 60 % had bronchopneumonia, and the most common symptoms were cough and fever.

Although known HPyVs cause disease in patients with immune-system imbalances, they do not seem to cause obvious illnesses in the great majority of infected individuals. However, BKPyV can induce nephropathy in kidney transplantation recipients and JCPyV causes progressive multifocal leukoencephalopathy. Progressively increasing or high viral loads are also associated with high-level viral replication and disease. For instance, progressive multifocal leukoencephalopathy and hemorrhagic cystitis are related to high viral loads of JCPyV and BKPyV, respectively. The present study cannot confirm that HPyV6 is the cause of RTIs in hospitalized children, because the viral loads of HPyV6 were low (1.38–182.42 copies/μl) and the coinfection rate with other respiratory viruses was high.

In previous reports, HPyVs were detected in the respiratory tract and skin but, to date, there has been insufficient evidence that HPyV6 is associated with any respiratory tract disease or skin disease [36]. Whether HPyV6 induce any disease requires the analysis of further data.

## Conclusion

The detection rate for HPyV6 by real-time PCR assay was 1.7 % in 887 NPA samples collected from hospitalized children with RTI. An association between HPyV6 and respiratory diseases could not been revealed due to the high coinfection rate and the low HPyV6 viral load.

## Methods

### Study population and samples

From 1 October, 2011, to 30 September, 2012, 887 nasopharyngeal aspirate (NPA) specimens were collected continuously from 887 hospitalized children with RTI, who ranged in age from 3 days to 14 years, at Beijing Friendship Hospital, Beijing, China. The patients’ parents or guardians gave their written informed consent for specimen collection and testing, and the project was approved by the Ethical Committee of the Beijing Friendship Hospital. The NPA specimens were collected and transported immediately to the National Institute for Viral Disease Control and Prevention, China CDC, and stored at −80 °C until further processing. All demographic data and clinical findings were recorded on a standard form. Total nucleic acids were extracted from each NPA specimen using the QIAamp MinElute Virus Spin Kit (Qiagen, Beijing, China).

### TaqMan real-time PCR assay

A TaqMan-based real-time PCR assay for the detection of VP1 gene of HPyV6 was designed with Primer Express 3.0 software. The primer sequences used were HPyV6-F 5’-TTAACACCCTTCTTTGTGCTGCTA-3’ and HPyV6-R 5’- GCCCAATTATTCAAAGCAGCTAA-3’, and the probe sequence was HPyV6-P FAM-CTGTCACAGGCCTGCTGAGCAATAGATTTC-TAMRA. The specificities of the primers and probe were evaluated in GenBank with BLAST. The primers and probe were synthesized by Invitrogen (Beijing, China). A common reaction mix was prepared for the real-time PCR assays. Briefly, the final 20 μl reaction mix contained 10 μl of TaqMan Gene Expression Master Mix, 1.8 μl of each primer (10 pmol/μl), 0.2 μl of probe (10 pmol/μl), 2 μl of pMD18-T/VP1 plasmid template (plasmid pMD18-T linked to the VP1 gene of HPyV6), and 4.2 μl of H_2_O. The amplification conditions included an initial incubation at 50 °C for 2 min and 95 °C for 15 min, followed by 40 cycles of 95 °C for 15 s and 60 °C for 1 min, using the Mx3005P qPCR System (Agilent Stratagene). Ten-fold serial dilutions of the pMD18-T/VP1 plasmid (from 10^0^ to 10^10^ copies/μl) were added to the real-time PCR reactions in duplicate. The results were used to generate a standard curve for HPyV6. Specificity was assessed by testing mixed samples of other common HPyVs, including WUPyV, KIPyV, JCPyV, BKPyV, and HPyV7. To test the reproducibility of the assay, we added 10^5^–10^8^ copies/μl of pMD18-T/VP1 plasmid to each reaction and each concentration of DNA was repeated five times. In addition, housekeeping gene glyceraldehyd-3-phosphate dehydrogenase (GAPDH) was used as internal control. 2 μl of nucleic acid of each NPA specimen was added to each reaction. Only samples that were positive according to both PCR and DNA sequencing were considered “positive”.

### Compiling the complete HPyV6 genome and phylogenetic analysis

Fifteen overlapping fragments of the complete genome of HPyV6 strain BJ376 were PCR amplified (Table 2) with the Takara Ex Taq kit. The 15 overlapping fragments were then cloned into pMD18-T and sequenced (Invitrogen). The nucleotide sequence of the full-length HPyV6 genome was then compiled using BioEdit 9.0 software. The full-length HPyV6 sequence was aligned with the sequences of other HPyVs and other HPyV6 strains available in GenBank with DNAStar software. A neighbor-joining tree was constructed with MEGA 6.0.

### Statistical analysis

The significance of differences between the prevalence rates and viral loads of various groups was tested with Fisher’s exact test and the Mann–Whitney *U* test. All analyses were performed with SPSS 19.0 software.

## Additional file

Additional file 1: Table S1. Primers and probes used to detect respiratory viruses. **Table S2.** Primers used to amplify HPyV6 with nested PCR (DOCX 28 kb)

## References

[1] Van Ghelue M, Khan MT, Ehlers B, Moens U (2012). Genome analysis of the new human polyomaviruses. Rev Med Virol.

[2] Dalianis T, Hirsch HH (2013). Human polyomaviruses in disease and cancer. Virology.

[3] Padgett BL, Walker DL, ZuRhein GM, Eckroade RJ, Dessel BH (1971). Cultivation of papova-like virus from human brain with progressive multifocal leucoencephalopathy. Lancet.

[4] Gardner SD, Field AM, Coleman DV, Hulme B (1971). New human papovavirus (B.K.) isolated from urine after renal transplantation. Lancet.

[5] Gaynor AM, Nissen MD, Whiley DM, Mackay IM, Lambert SB, Wu G (2007). Identification of a novel polyomavirus from patients with acute respiratory tract infections. PLoS Pathog.

[6] Allander T, Andreasson K, Gupta S, Bjerkner A, Bogdanovic G, Persson MA (2007). Identification of a third human polyomavirus. J Virol.

[7] Feng H, Shuda M, Chang Y, Moore PS (2008). Clonal integration of a polyomavirus in human Merkel cell carcinoma. Science.

[8] Schowalter RM, Pastrana DV, Pumphrey K, Moyer AL, Buck CB (2010). Merkel cell polyomavirus and two previously unknown polyomaviruses are chronically shed from human skin. Cell Host Microbe.

[9] Van der Meijden E, Janssens RW, Lauber C, Bouwes Bavinck JN, Gorbalenya AE, Feltkamp MC (2010). Discovery of a new human polyomavirus associated with trichodysplasia spinulosa in an immunocompromized patient. PLoS Pathog.

[10] Scuda N, Hofmann J, Calvignac-Spencer S, Ruprecht K, Liman P, Kuhn J (2011). A novel human polyomavirus closely related to the african green monkeyderived lymphotropic polyomavirus. J Virol.

[11] Buck CB, Phan GQ, Raiji MT, Murphy PM, McDermott DH, McBride AA (2012). Complete genome sequence of a tenth human polyomavirus. J Virol.

[12] Yu G, Greninger AL, Isa P, Phan TG, Martínez MA, Sanchez M (2012). Discovery of a novel polyomavirus in acute diarrheal samples from children. PLoS One.

[13] Siebrasse EA, Reyes A, Lim ES, Zhao G, Mkakosya RS, Manary MJ (2012). Identification of MW polyomavirus, a novel polyomavirus in human stool. J Virol.

[14] Lim ES, Reyes A, Antonio M, Saha D, Ikumapayi UN, Adeyemi M (2013). Discovery of STL polyomavirus, a polyomavirus of ancestral recombinant origin that encodes a unique T antigen by alternative splicing. Virology.

[15] Korup S, Rietscher J, Calvignac-Spencer S, Trusch F, Hofmann J, Moens U (2013). Identification of a novel human polyomavirus in organs of the gastrointestinal tract. PLoS.

[16] Mishra N, Pereira M, Rhodes RH, An P, Pipas JM, Jain K (2014). Identification of a novel polyomavirus in a pancreatic transplant recipient with retinal blindness and vasculitic myopathy. J Infect Dis.

[17] Bogdanovic G, Priftakis P, Giraud G, Kuzniar M, Ferraldeschi R, Kokhaei P (2004). Association between a high BK virus load in urine samples of patients with graftversus-host disease and development of hemorrhagic cystitis after hematopoietic stem cell transplantation. J Clin Microbiol.

[18] Shishido-Hara Y (2010). Progressive multifocal leukoencephalopathy and promyelocytic leukemia nuclear bodies: a review of clinical, neuropathological, and virological aspects of JC virus-induced demyelinating disease. Acta Neuropathol.

[19] Shah KV (2007). SV40 and human cancer: a review of recent data. Int J Cancer.

[20] Kean JM, Rao S, Wang M, Garcea RL (2009). Seroepidemiology of human polyomaviruses. PLoS Pathog.

[21] Bialasiewicz S, Whiley DM, Lambert SB, Jacob K, Bletchly C, Wang D (2008). Presence of the newly discovered human polyomaviruses KI and WU in Australian patients with acute respiratory tract infection. J Clin Virol.

[22] Debiaggi M, Canducci F, Brerra R, Sampaolo M, Marinozzi MC, Parea M (2010). Molecular epidemiology of KI and WU polyomaviruses in infants with acute respiratory disease and in adult hematopoietic stem cell transplant recipients. J Med Virol.

[23] Templeton KE, Scheltinga SA, Beersma MF, Kroes AC, Claas EC (2004). Rapid and sensitive method using multiplex real-time PCR for diagnosis of infections by influenza A and influenza B viruses, respiratory syncytial virus, and parainfluenza viruses 1, 2, 3, and 4. J Clin Microbiol.

[24] McLeish NJ, Witteveldt J, Clasper L, McIntyre C, McWilliam Leitch EC, Hardie A (2012). Development and Assay of RNA Transcripts of Enterovirus Species A to D, Rhinovirus Species A to C, and Human Parechovirus:Assessment of Assay Sensitivity and Specificity of Real-Time Screening and Typing Methods. J Clin Microbiol.

[25] Kuypers J, Campbell AP, Guthrie KA, Wright NL, Englund JA, Lawrence C (2012). WU and KI polyomaviruses in respiratory samples from allogeneic hematopoietic cell transplant recipients. Emerg Infect Dis.

[26] Bialasiewicz S, Whiley DM, Lambert SB, Gould A, Nissen MD, Sloots TP (2007). Development and evaluation of real-time PCR assays for the detection of the newly identified KI and WU polyomaviruses. J Clin Virol.

[27] Kim JS, Lim CS, Kim YK, Lee KN, Lee CK (2011). Human bocavirus in patients with respiratory tract infection. Korean J Lab Med.

[28] Heim A, Ebnet C, Harste G, Pring-Akerblom P (2003). Rapid and quantitative detection of human adenovirus DNA by real-time PCR. J Med Virol.

[29] Maertzdorf J, Wang CK, Brown JB, Quinto JD, Chu M, de Graaf M (2004). Real-time reverse transcriptase PCR assay for detection of human metapneumoviruses from all known genetic lineages. J Clin Microbiol.

[30] Ward CL, Dempsey MH, Ring CJ, Kempson RE, Zhang L, Gor D (2004). Design and performance testing of quantitative real time PCR assays for influenza A and B viral load measurement. J Clin Virol.

[31] Esposito S, Bosis S, Niesters HG, Tremolati E, Begliatti E, Rognoni A (2006). Impact of human coronavirus infections in otherwise healthy children who attended an emergency department. J Med Virol.

[32] Antonsson A, Bialasiewicz S, Rockett RJ, Jacob K, Bennett IC, Sloots TP (2012). Exploring the prevalence of ten polyomaviruses and two herpes viruses in breast cancer. Plos One.

[33] Scola N, Wieland U, Silling S, Altmeyer P, Stücker M, Kreuter A (2012). Prevalence of human polyomaviruses in common and rare types of non-Merkel cell carcinoma skin cancer. Br J Dermatol.

[34] Rockett RJ, Sloots TP, Bowes S, O’Neill N, Ye S, Robson J (2013). Detection of novel polyomaviruses, TSPyV, HPyV6, HPyV7, HPyV9 and MWPyV in feces, urine, blood, respiratory swabs and cerebrospinal fluid. PLoS One.

[35] Siebrasse EA, Bauer I, Holtz LR, Le B-M, Lassa-Claxton S, Canter C (2012). Human polyomaviruses in children undergoing transplantation, United States, 2008–2010. Emerg Infect Dis.

[36] Schrama D, Buck CB, Houben R, Becker JC (2012). No evidence for association of HPyV6 or HPyV7 with different skin cancers. J Invest Dermatol.

