# Supplementary material for: Human polyomavirus type six in respiratory samples from hospitalized children with respiratory tract infections in Beijing, China
Source: Virol J. 2015 Oct 13;12:166. doi: 10.1186/s12985-015-0390-5 (PMC4604616; doi:10.1186/s12985-015-0390-5)
Supplement: Additional file 1: Table S1. — Primers and probes used to detect respiratory viruses. Table S2. Primers used to amplify HPyV6 with nested PCR (DOCX 28 kb) [file 12985_2015_390_MOESM1_ESM.docx]

**Supplementary Table 1. Primers and probes used to detect respiratory viruses.**

| **Viruses detected** | **primer and probe** | **Sequence（5′-3′）** | **Target genes** | **Ref** |
| --- | --- | --- | --- | --- |
| **RSV** | RSVF | TTTCCACAATATYTAAGTGTCAA | M | 23 |
|  | RSVR | TCATCWCCATACTTTTCTGTTA |  |  |
|  | RSVPb | FAM-GCGAGCCCATGTGAATTCCCTGCATCAATGCTCGC-BHQ1 |  |  |
| **HRV** | HRVF | A1:AGCCTGCGTGGCTGCCTG /A2: CCTGCGTGGCGGCCARC | UTR | 24 |
|  | HRVR | CCCAAAGTAGTYGGTCCCRTCC |  |  |
|  | HRVPb | FAM-TCCTCCGGCYCCTGAATG-MGBNFQ |  |  |
| **KIPyV** | KIF | CTATCCCTGAATACCAGTTGGAAAC | VP2-3 | 25 |
|  | KIR | GTATGACGCGACAAGGTTGAAG |  |  |
|  | KIPb | FAM-TTCCGGGCATCCCAGACTGGC-BHQ1 |  |  |
| **WUPyV** | WUF | CTACTGTAAATTGATCTATTGCAACTCCTA | LTAg | 26 |
|  | WUR | GGGCCTATAAACAGTGGTAAAACAACT |  |  |
|  | WUPb | FAM-CCTTTCCTCCACAAAGGTCAAGTAAA-TAMRA |  |  |
| **HBoV** | HBoVF | GCAAATCTCTTCTGGCTACACG | NS-1 | 27 |
|  | HBoVR | CCTCTGCGATCTCTATATTGAAGG |  |  |
|  | HBoVPb | FAM-ATGTTGCCGCCAGTAACTCCACCC-TAMRA |  |  |
| **ADV** | ADVF | GCCACGGTGGGGTTTCTAAACTT | Hexon | 28 |
|  | ADVR | GCCCCAGTGGTCTTACATGCACATC |  |  |
|  | ADVPb | FAM-TGCACCAGACCCGGGCTCAGGTACTCCGA-TAMRA |  |  |
| **hMPV** | hMPVF | CATATAAGCATGCTATATTAAAAGAGTCTC | N | 29 |
|  | hMPVR | CCTATTTCTGCAGCATATTTGTAATCAG |  |  |
|  | hMPVPb | FAM-TGYAATGATGAGGGTGTCACTGCGGTTG-TAMRA |  |  |
| **IFVA** | IFVAF | AAGACCAATCCTGTCACCTCTGA | M1 | 30 |
|  | IFVAR | CAAAGCGTCTACGCTGCAGTCC |  |  |
|  | IFVAPb | FAM-TTTGTGTTCACGCTCACCGT-TAMRA |  |  |
| **IFVB** | IFVBF | GAGACACAATTGCCTACCTGCTT | M | 31 |
|  | IFVBR | TTCTTTCCCACCGAACCAAC |  |  |
|  | IFVBPb | FAM-AGAAGATGGAGAAGGCAAAGCAGAACTAGC-TAMRA |  |  |
| **PIV-1** | PIV1F | ACCTACAAGGCAACAACATC | HN | 23 |
|  | PIV1R | CTTCCTGCTGGTGTGTTAAT |  |  |
|  | PIV1Pb | FAM- GCTGCCCAAACGATGGCTGAAAAAGGGAGGCAGC-TAMRA |  |  |
| **PIV-2** | PIV2F | CCATTTACCTAAGTGATGGAA | HN | 23 |
|  | PIV2R | CGTGGCATAATCTTCTTTTT |  |  |
|  | PIV2Pb | FAM-GCTGCCAATCGCAAAAGCTGTTCAGTCACGGCAGC-TAMRA |  |  |
| **PIV-3** | PIV3F | GGAGCATTGTGTCATCTGTC | HN | 23 |
|  | PIV3R | TAGTGTGTAATGCAGCTCGT |  |  |
|  | PIV3Pb | FAM-CGCGCTACCCAGTCATAACTTACTCAACAGCAACAGCGCG-TAMRA |  |  |
| **PIV4** | PIV4 F | CCTGGAGTCCCATCAAAAGT | HN | 23 |
|  | PIV4 R | GCATCTATACGAACACCTGCT |  |  |
|  | PIV4 Pb | FAM-GCTGCCGTCTCAAAATTTGTTGATCAAGACAATACAATTGGCAGC-BHQ2 |  |  |
| **HCoV-229E** | 229EF | CGCAAGAATTCAGAACCAGAG | N | 31 |
|  | 229ER | GGCAGTCAGGTTCTTCAACAA |  |  |
|  | 229EPb | FAM-CCACACTTCAATCAAAAGCTCCCAAATG-TAMRA |  |  |
| **HCoV-OC43** | OC43F | GCTCAGGAAGGTCTGCTCC | N | 31 |
|  | OC43R | TCCTGCACTAGAGGCTCTGC |  |  |
|  | OC43Pb | FAM-TTCCAGATCTACTTCGCGCACATCC-TAMRA |  |  |
| **HCoV-HKU1** | HKU1F | AGTTCCCATTGCTTTCGGAGTA | N | 31 |
|  | HKU1R | CCGGCTGTGTCTATACCAATATCC |  |  |
|  | HKU1Pb | FAM-CCCCTTCTGAAGCAA-MGB |  |  |
| **HCoV-NL63** | NL63F | AGGACCTTAAATTCAGACAACGTTCT | N | 31 |
|  | NL63R | GATTACGTTTGCGATTACCAAGACT |  |  |
|  | NL63Pb | FAM-TAACAGTTTTAGCACCTTCCTTAGCAACCCAAACA-TAMRA |  |  |

**Supplementary Table 2. Primers used to amplify HPyV6 with nested PCR.**

| **Number** | **Primer sequences （5′-3′）** | **Amplicon size（bp）** |
| --- | --- | --- |
| **1** | TTTATCCAGTGAAGCAACT | 377 |
|  | CCTATTTCCTCTCCTTTTT |  |
|  | GTTCCTGAGTTGTCCCA |  |
|  | GCCTTTATTGCCTCCTA |  |
| **2** | AGAGGCCCTGGAAATTGCTGGT | 403 |
|  | TCCATAACCTCCACTCCTCCTT |  |
|  | CAGTCTGCTGCTGCTTTATCC |  |
|  | TTATCTCCCTGCCTGCTCTAT |  |
| **3** | CGACTGGCTTATCAACAT |  |
|  | CAGCTCCTCAAGAACAAA | 393 |
|  | CGACTGGCTTATCAACAT |  |
|  | CTTACCCAAAGACCTTCA |  |
| **4** | CATTGTCAGAGGAATATG | 399 |
|  | AAGTGTCAGTAAAGGTGT |  |
|  | CTGTTTGAGGGCAATGGAGTA |  |
|  | GTGTCAGTAAAGGTGTAGGGG |  |
| **5** | ATGGCTGCTTTTTGTTC | 295 |
|  | TGGAGTGGTGCTCTGAT |  |
|  | CCCCTACACCTTTACTG |  |
|  | TGCTACATTCATTCTTT |  |
| **6** | GCTGGTTCATCTCTGTGTTAT | 415 |
|  | TAGTTGGAAGTATGCTTGGCT |  |
|  | TATAAGAGCACAAGGAACCCC |  |
|  | ATACCCTACCATCCCCAACAT |  |
| **7** | AATTTGGAGTGCTGATCCT | 436 |
|  | TTCCTTGCTTGTCTGTTTC |  |
|  | ATTTTGGCAGAATTGTTGGTGG |  |
|  | AGAATAAACTTTGTACTGGGTG |  |
| **8/9** | TGTTGGGGATGGTAGGGTATG |  |
|  | GAGCCAGTGGAGGACTTTATG |  |
| **8** | GCTGGTAACCCCACTCTCA | 489 |
|  | GCTCCATGCATTCCATGTA |  |
| **9** | AGAAACAGACAAGCAAGGAAC | 333 |
|  | ATGGAAGAACTGCAGGAAGAT |  |
| **10** | AGAAACAGACAAGCAAGGAAC | 613 |
|  | TATGCAGGAGGTGTGGAAATT |  |
|  | TCATTTGTTTCATCATTTTCC |  |
|  | ATCTTGTAGGGGGTGTGTCTT |  |
| **11** | CAATGTTGTTTTGCCACTA | 317 |
|  | ATGGGAAACCTTGTATGAT |  |
|  | AGACACACCCCCTACA |  |
|  | ATGCCCAATTATTCAAACA |  |
| **12** | TTTCCACACCTCCTG | 422 |
|  | ACTTTCCATCTTGCC |  |
|  | CTTCTTTGTGCTGCTACTCTGT |  |
|  | GTGTCTGCTATGCTAAATGCTT |  |
| **13** | TCCAATCACATTCCTCTTT | 450 |
|  | GGACCCACATTATCTACCA |  |
|  | ATCACATTCCTCTTTTTTGG |  |
|  | AAGTATTCTTGCACTCCTCC |  |
| **14** | TTGGAGCAGGATTGGGTTTT | 474 |
|  | AGGTCAGTGATGATTGGGGT |  |
|  | TGCTGTCTTGAGAGTTGCC |  |
|  | GATTTCCGTTTGTGTGTAC |  |
| **15** | GTAGCACTTGTAGCACCAGCACTTT | 509 |
|  | ACTAATTAGGGTGTTGTGTCAACGG |  |
|  | GTTGTACACACAAACGGAAATC |  |
|  | GCCCTATTTCCTCTCCTTTTTT |  |

Note: The first pair of primers is the outer pair, and the second pair of primers is the inner pair in every group;

nos 8 and 9 have the same outer primer pair.
